# Potential anticancer activity of Acetone extracts of *Toona cilliata*, *Seriphium plumosum* and *Schkuhria pinnata* on HeLa cervical cancer cells

**DOI:** 10.4314/ahs.v21i2.23

**Published:** 2021-06

**Authors:** Mxolisi Justice Ndlovu, Victor Patrick Bagla, Matlou Phenius Mokgotho, Marema Ephraim Makgatho, Thabe Moss Matsebatlela

**Affiliations:** 1 Department of Biochemistry, Microbiology and Biotechnology Faculty of Sciences and Agriculture; 2 Department of Pathology and Medical Sciences, Faculty of Health Sciences,University of Limpopo, South Africa

**Keywords:** Medicinal plants, anticancer activity, antioxidant activity

## Abstract

**Background:**

Cervical cancer is common in women in less developed regions of the world. The plant biomolecules can be employed for synergistic activity with chemo- and radiotherapy. This combinations might result in reduced toxicity and increased efficacy of the treatment regimen.

**Objectives:**

The anti-HeLa cells activity of the acetone extracts of *S. plumosum*, T. cilliata and *S. pinnata* was assessed using different parameters.

**Methods:**

Secondary metabolite detection and antioxidant activity quantification were determined using the DPPH and ferric iron reducing assays. HeLa cell growth inhibition and mechanistics were assessed by employing MTT and Annexin-V flous assays.

**Results:**

Observations revealed the presence of phenolic, flavonoids, tannins steroids and coumarins in all the plants extracts. High amount of total phenolic and flavonoid content were detected in *S. plumosum* and *T. cilliata. S. plumosum* extract had the best DPPH scavenging activity and ferric reducing powers.

**Conclusion:**

Observable concentration dependent cell proliferation inhibition by test materials was exhibited. The leaf extracts from T. cilliata, *S. plumosum* and *S. pinnata* contain compounds of various polarities with free-radical, antioxidant and anti-cancerous activities that may play a beneficial role in treatment.

## Introduction

The prevalence of cervical cancer has reached alarming global levels where in 2010 it was reported to be 4-fold higher prevalence of cervical cancer in low to middle-income countries^1^. Countries ranked low in the Human Development Index (HDI), the disease is ranked as the second most common type of cancer and second highest cause of cancer-related mortality amongst women after breast cancer. In Africa, cervical cancer is the most diagnosed and the leading cause of cancer-related death among^2^. The disease affects one in 42 women and available reports from Statistics South Africa (2014) estimates that 16.84 million women over the age of 15 are at risk of the disease. At present, more than 8 South African women die from the disease every day^3^. This has prompted the observance in the month of September of each year as the cervical cancer awareness month in South Africa, to highlight the impact of the disease. Although vaccines are available for the prevention of the disease and can be administrated to girls between 9 and 13 years, they are unaffordable^3^.

Immunization has been considered to unlikely generate significant therapeutic effects for establishing antibody-mediated neutralization^4^. Therapeutic agents with fewer side effects can serve to bridge this gap. The plants under investigation were selected based on their traditional indication in the treatment of patients exhibiting symptoms of cervical cancer by traditional healers in Limpopo Province of South Africa. *T. cilliata* is reported to be used mostly on chronic dysentery, ulcer and blood complaints as a cardiotonic, antihelmintic and as an expectorant^5^. *S. pinnata* is used mainly for blood cleansing, malaria and urinary tract infections^6^, while *S. plumosum* is used mainly for heart problems, epilepsy and postural realignment^7^. The present study focused on the anti-proliferative effects of acetone extracts of selected medicinal plants on proliferation of a HeLa cancer cell line, antioxidant activity, secondary metabolites, phytochemical constituencies and apoptotic effects.

## Materials and Methods

**Plant collection and verification of leaves.**

*S. plumosum* and *S. pinnata* were collected from Mankweng and *T. cilliata* from Tzaneen in Limpopo province, South Africa. Plants were identified by the Larry Leach Herbarium, University of Limpopo and vouchered as *S. plumosum* (UNIN 121065), T. cilliata (UNIN 12331) and *S. pinnata* (UNIN 121066).

**Plant extracts preparation**. The leaves of the plants were dried in the dark at 250C for two weeks and grinded into fine powder (1g) which was exhaustively extracted using 10 ml of acetone8. The supernatants were filtered into pre-weighed glass vials. The quantity of plant materials extracted was determined and stored in air-tight glass vials in the dark until use. The dry plant extracts were reconstituted in dimethylsulphoxide (DMSO) (Sigma Aldrich, SA) for cell-based assays.

**Phytochemical profiling of plant material.**

Extracts of the selected plants were reconstituted in acetone (1 mg/ml) and spotted on aluminium backed thin layer chromatography plates (TLC) (Macherey-NEGEL, GMBH & CO.KG) for analysis. Each extract (10 µl) was loaded onto a TLC plate and developed in three different mobile phase viz: Chloroform: Ethyl acetate: Formic acid [CEF] 10:8:2 (v/v/v) (intermediate polarity/acidic), Ethyl acetate: Methanol: Water [EMW] 10:1, 35:1 (v/v/v) (polar/neutral) and Toluene: Ethanol: Ammonium hydroxide [TEA] 18:2:0.2 (v/v/v) (non-polar/basic). The separated compounds were visualized under ultraviolet light at wavelengths of 254 and 360nm. The plates were sprayed with vanillin-sulphuric acid (0.1 g vanillin (sigma): 28 ml methanol) and heated at 1100C to visualize coloured bands on the plates.

**Qualitative antioxidant analysis using DPPH**.

Thin layer chromatographic plates were prepared as described for in the previous section and sprayed with 0.2% of 2.2- 2, 2-diphenyl-1-picrylhydrazyl (DPPH) in methanol solution to detect the presence of compounds with antioxidant activity. A positive result was represented by the presence of yellow bands on a purple background and scanned using a scanner (CanoScan Lide 700F)^9^.

**Quantitative antioxidant activity assay (DPPH assay)**

The plant extracts were tested for scavenging activity using quantitative methods as described by^10^. Thin layer chromatography (TLC) plates were used to separate compounds of the extracts with antioxidant activity. The 2, 2- diphenyl-1-picrylhydrazyl (DPPH) indicator, was sprayed on the plates to assist in viewing the antioxidant compounds. The quantitative method was performed using micro-titer plates, where 100 µl of each plant extract was serially diluted with water and was tested for antioxidant activity at various concentrations. DPPH (100 µl) was added into the wells after dilution. Ascorbic acid (vitamin C) was used as positive control and absorbance was read at 540 nm using M8500 UV-Visible spectrophotometer made in China.

**Qualitative screening of various phytochemical constituencies from the selected medicinal plants**

Phenols. The presence of phenols was indicated by a blueish and greenish colour change after additional of test material and distilled water^11^.

**Tannins.** After the addition of the plant extract and distilled water as well as ferric chloride, the presence of a greenish-black colour was indicative of tannins^12^.

**Flavonoids.** Flavonoids tested positive due a yellow colour after adding the test material, ammonia solution and concentrated H2SO4^13^.

**Steroids.** The Salkowski's test was used^14^.

Coumarins. A total volume of 2 ml of the extract was mixed with 3 ml of NaOH (10%), a yellow coloration was observed to draw an inference, indicating the presence of coumarins^15^.

**Determination of total phenolic content.**

The total phenolics in extracts was determined spectrophotometrically using Folin-Ciocalteu`s phenol reagent. Garlic acid was used as a standard and the total phenolics were expressed as mg/g gallic acid equivalence (GAE). Concentration of 0.01, 0.02, 0.03 0.04 and 0.05 mg/ml of gallic acid were prepared in methanol. Concentration of 0.1 and 1mg/ml of plant extracts were also prepared in methanol and 0.5ml of each sample were introduced into the test tubes and mixed with 2.5 ml of a 10- fold dilute Folin- Ciocalteu reagent and 2 ml of 7.5% sodium carbonate. The tubes were covered with parafilm and allowed to stand for 30 minutes at 250C before the absorbance was read at 760 nm using (Beckman Coulter DU® 730 and Life Science UV-Visible spectrophotometer). All determinations were performed in triplicate. Folin-Ciocalteu reagent being using sensitive to reducing compounds including polyphenols produce a blue color upon reaction which was measured spectrophotometric^16^. (Beckman Coulter DU® 730 and Life Science UV-Visible spectrophotometers was used) South Africa.

**Determination of total flavonoids.** The presence of flavonoids in the plant extracts was determined using aluminium chloride colorimetric assay as described by17. Volume of 1 ml of plant extract was mixed with 4 ml of distilled water and subsequently with 0.30 ml of a NaNO2 solution (10%). After 5 minutes, 0.30 ml solution AICI3 (10%) was added followed by 2 ml of 1% NaOH solution to the mixture. Immediately, the mixture was thoroughly mixed and absorbance was determined at 510 nm versus the blank using T60 UV- Visible spectrophotometer (Roche, S.A). Standard curve of quercetin was prepared (0–12 mg/ml) and the results were expressed as quercetin equivalents (mg quercetin/gm dried extract).

**Cell culture.** An immortalised human cell line (HeLa) was used in this study (ATCC® CCL-2™, Rockville, USA). The cells were cultured and maintained in RPMI media (Lonza, BioWhittaker®), supplemented with 10% foetal bovine serum (Hyclone, Thermo Scientific) at 37°C, in an atmosphere of 5% CO_2_ in a humidified incubator (Heracell 150i CO_2_ incubator, Thermo Scientific)

**Determination of the effects of plant extracts on HeLa cell proliferation using the MTT assay.**

HeLa cells were seeded in 96 well tissue culture plates at a density of 2 x 10^4^ cells/ml and treated with various concentrations (50 – 1000µg/ml) of extract for 24 hours. After 24 hours of incubation, MTT solution (2.5 mg/ml) was added to each well (20 ml). The cells were incubated for a further 4 hours at 37°C, and the supernatant was removed. The pallet was then dissolved by addition of 100 µl of DMSO. The antiproliferative activity of extracts was determined following the conversion of tetrazolium by the enzyme dehydrogenase to purple formazan19 which directly represent the number of viable cells. The absorbance was measured at 560 nm using a Glomax microtiter plate (Promega, U.S.A). Actinomycin D was used as positive control while untreated cells where used as negative control. Percent cell viability was calculated using the formula: Percentage viability = (A490nm of sample) x 100)/ (A490nm of control)

**Determination of apoptotic activity effects of the selected plant extracts on HeLa cells.**

The cells were seeded over a cover slip and put inside a six well plate for 24 hours. The cells were then treated and incubated for 24 hours in a CO2 incubator. The slips were washed followed by addition of paraformaldehyde and incubated for 30 minutes. After incubation it was followed by a washing step and addition of Annexin V FICT solutions for 30 minutes. This was followed by viewing under fluorescent of X60 magnification with Nixon Ti- E inverted microscope (Nikon Japan) using different excitations.

## Results

**Figure 1:**
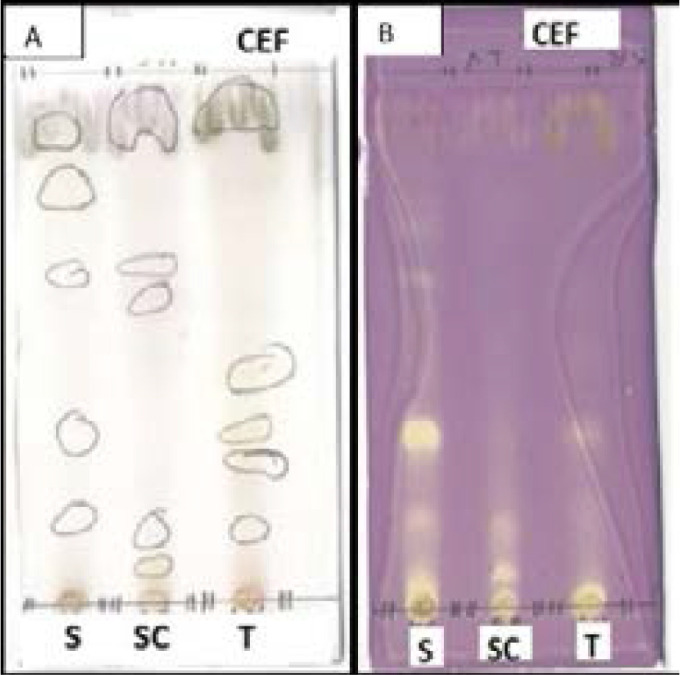
Thin layer chromatograms of acetone leaf extracts obtained from S. *plumosum* (S)*, S. pinnata* (SC) and *T. cilliata* (T) developed in CEF as mobile phases. The chromatograms were sprayed with vanillin/H_2_SO_4_ (A) and DPPH for detection of antioxidant compounds (B).

Extraction yield, TLC profile and qualitative antioxidant activity of acetone leaf extracts. The plant leaf materials (10 g) exhaustively extracted using 100 ml of acetone yielded 0,0432g for *S. plumosum, T. cilliata* (0,1786g) and *S. pinnata* (0,0295g). A representative fingerprint profile of the three acetone extracts in the eluent system (CEF) that best separated compounds with antioxidant activity is presented (figure 1). Antioxidant activity profile show *S. plumosum* to contain more compounds with antioxidant activity as compared to the other two plant extracts (figure 1B).

Evaluating the presence of secondary metabolites in the acetone extracts of the selected medicinal plants

### Determination of Phenolic contents of the acetone extracts of the selected medicinal plants

The total phenolics in extracts were determined (figure 2) using Garlic acid as a standard and expressed as mg/ml gallic equivalence (GAE).

**Figure:**
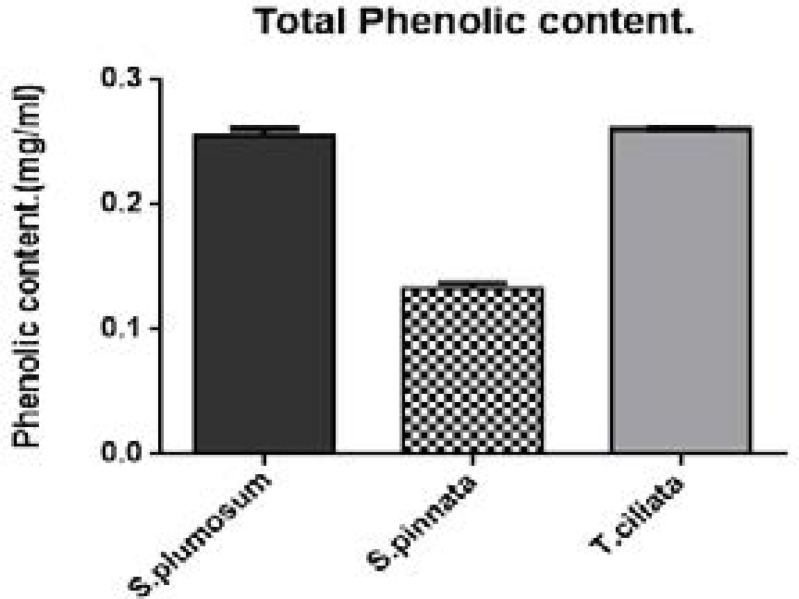


### Determination of total flavonoids contents of the acetone extracts of the selected medicinal plants

**Figure 3:**
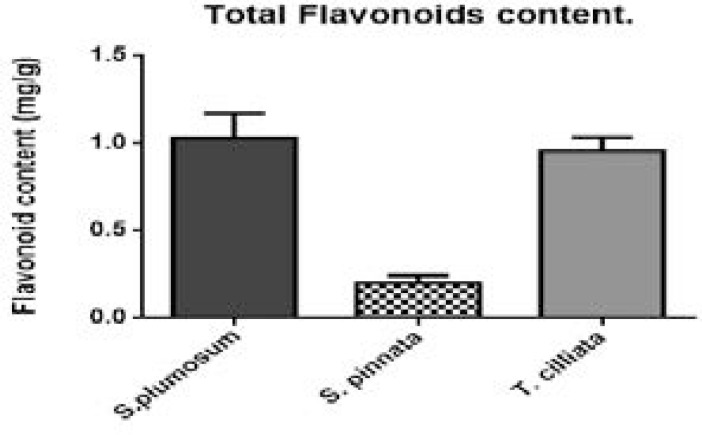
Total flavonoids content

The flavonoid content of extracts was measured by the aluminum chloride colorimetric assay (figure 3).

Cell proliferation determination of the acetone leaf extracts of the selected medicinal plants

### Determination of apoptotic effects of the selected plant extracts

**Figure 7:**
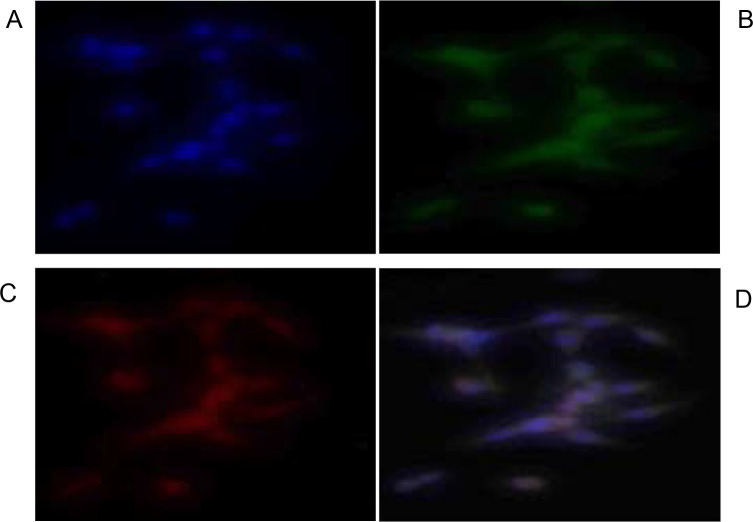
Apoptotic effects

**Figure 8:**
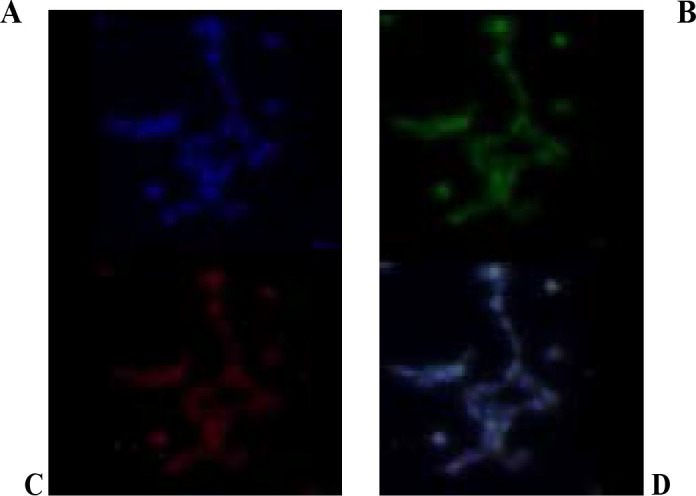
Apoptotic effects of *T. cilliata* acetone leaf extract. Cells were cultured at a density of 6x106 cells/ml, on coverslip in micropetridishes. **(A)** represents a slide incubated with 4′,6-diamidino-2-2 phenylindole (DAPI), **(B)** represents a slide incubated after adding Annexin V Fluos and shows the flipping of phosphatidylserine, **(C)** represents the disintegrated nucleus after the movement of annexin V into the cell and **(D)** represents the slide after overlaying with all the colours.

**Figure 9:**
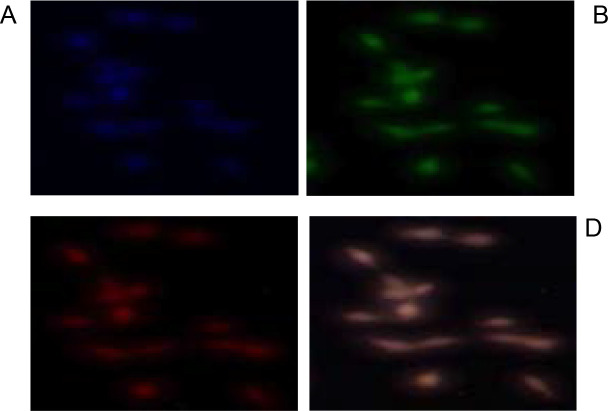
Apoptotic effects

Annexin-V and PI apoptosis detection assay was used to test for the ability of the acetone leaf extracts of *S. plumosum, T. cilliata* and *S. pinnata* to induce apoptosis (figure 7, 8 and 9).

## Discussion

Cervical cancer is one of the detrimental and life-threatening female cancers with its early stages being cured with surgery and a combination of chemotherapy (cisplatinum) and radiation therapy. Thus, treatment that is effective, less toxic, affordable is desirable. Thus, the current study was therefore aimed at evaluating the extracts of the selected plants to induce apoptosis. Acetone was the choice in this study due to its ability to extract both polar and non-polar constituents.

Phytochemical analysis of the plant extracts revealed the presence of phenolics, flavonoids, tannins, steroids and coumarins in all the plants. Phenolic compounds are one of the largest and common groups of plant metabolites^18^. They have important biological properties such as anti-carcinogenic, anti-inflammatory, as well as the inhibition of the angiogenesis process^19^. All the plants tested positive for phenols which exhibit antioxidant and antiproliferative tumor effects whereas ethanolic extracts of *S. pinnata* have been reported to exhibit anticancer activity against a human colon cancer cell line (Caco-2)^20^. Flavonoids have been reported for playing an important role in preventing carcinogenesis through anti-proliferation, anti-oxidation and apoptosis activity in various cancer cell lines^21^, Tannic acid possible application versatility not only in cancer prophylaxis, as was initially thought, but also in adjuvant cancer therapy was also reported^22^. With steroids there was increasing research attention through investigating the novel theapeutic potential of steroidal cardiac glycosides in the past decade^23^. Curcumin has also been reported to have anti-cancer activity in both non-small cell lung and small cell lung cancer^24^. On the other hand, this study represents the first report of the anticancer activity of *T. cilliata* and *S. plumosum*. The detected phytochemical compounds contained in these plants have immersed bioactive activities and can be considered to be valuable reservoir of bioactive compounds of a high medicinal good.

These compounds, however, still need to be isolated and identified. The ferric reducing antioxidant power assay also showed an increase in percentage ferric iron reduction with increase in the concentration of the extracts and the positive control. This finding, furthermore, supported the antioxidant activities of the plant extracts. Toxicological studies were conducted on the plants to examine the toxicity profiles of these plant extract constituents to produce safe and less toxic new drugs.

Determination of apoptotic effects of the acetone leaf extracts was performed against cervical cancer HeLa cells. The method used in this study, is based on the degradation of the nucleus, condensation of the chromatin and flipping of the cell membrane^24^. Following treatment of cells with 400 µg/ml of the different plant extracts, the cells were shown to have undergone apoptosis, this concentration was chosen because it was almost the intermediate of the highest concentration. The heavily stained nucleus in (A) represents the nuclei considered to have the abnormal phenotypSec. hoAlasrOdneed, u37c5eGdreferonbmrietrhDerivfieg, uCrhearslo, tSte.svpilllue,mVAo,s2u2m901showed to have the highest concentration of the stain followed by *T. cilliata* and lastly *S. pinnata* which shows that it induced more cytotoxicity effects against the cell line.

Propidium iodide is a cell permeant fluorochrome that stains the DNA and is used to identify the mode of cell death and phase of apoptosis pictures (B). The Annexin V- FITC green fluorescence is used to identify the mode of cell death through flipping of the membrane. The fluorescence intensity in (figure 7, 8 and 9) represent the phase and amount of cell undergoing apoptosis. The higher red fluorescence represents cell death through the disintegrations of the nucleus after the movement of annexin V into the cell, while (D) represents fluorescence after overlaying all the three images. After overlaying the images, cells displaying simultaneous reddish nuclear and a greenish membrane fluorescence, represent cells that have undergone apoptosis^20^. Apoptosis is important since it does not promote continuous growth of cells that finally leads to cancer.

## Conclusion

All leaf extracts were shown to contain various phytochemical constituents. S. plumosum showed more antioxidant activity as compared to the other extracts. All leaf extracts were shown to contain secondary metabolites such as Phenolics, flavonoids, tannins, steroids and coumarins. All the plant extracts were cytotoxic to HeLa cells. Studies will be ongoing to determine the effect of the extracts on the expression underlying mechanisms in cervical cancer progression with the aim of identifying therapeutic principles contained in the extracts for the treatment of cervical cancer.

## Figures and Tables

**Figure 4:**
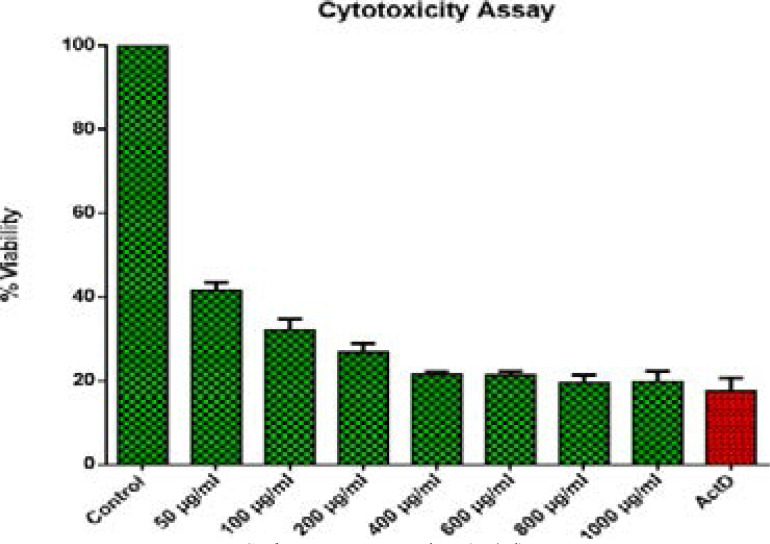
Cytotoxicity effects of *S. plumosum* acetone leaf extract. HeLa cells at a density of 1x 10^6^ were incubated for 24 hours and treated with different concentrations ranges of 50 µg/ml - 1000 µg/ml. The results are presented as ± SEM from triplicate of three independent experiments. *ActD stands for Actinomycin D.

**Figure 5:**
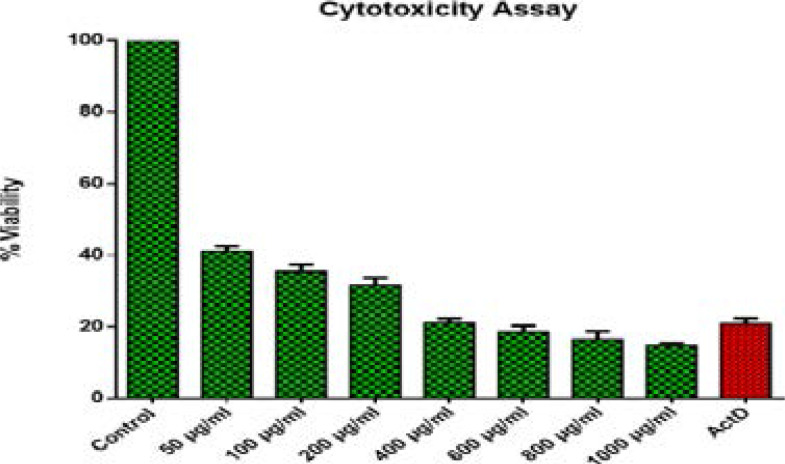
Cytotoxicity effects of *T. cilliata* acetone leaf extract. HeLa cells at a density of 1x 10^6^ were incubated for 24 hours and treated with different concentrations ranges of 50 µg/ml - 1000 µg/ml. The results are presented as ± SEM from triplicate of three independent experiments. *ActD stands for Actinomycin D.

**Figure 6:**
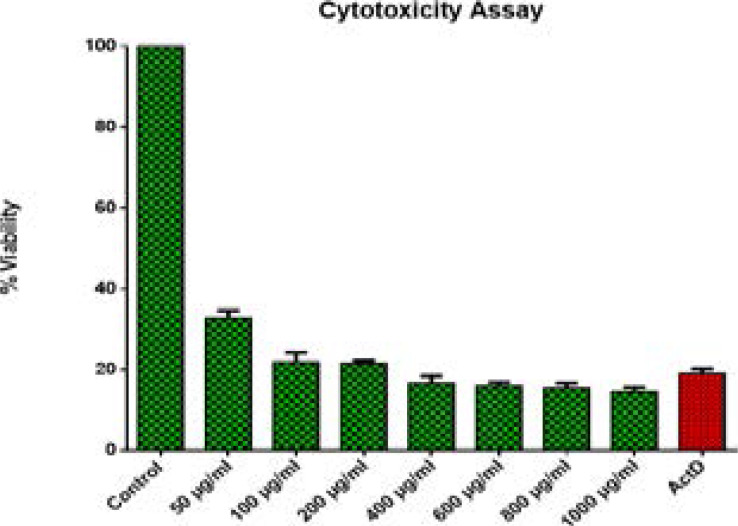
Cytotoxicity effects of *S. pinnata* acetone leaf extract. HeLa cells at a density of 1x 10^6^ were incubated for 24 hours and treated with different concentrations ranges of 50 µg/ml - 1000 µg/ml. The results are presented as ± SEM from triplicate of three independent experiments. *ActD stands for Actinomycin D.

**Table 1:** Types of secondary metabolites detected in the acetone leaf extracts of *S. plumosum, S. pinnata* and *T. cilliata* using chemical methods

	Type of extract		

Secondary metabolites	S	SC	T
Phenolics	+	+	+
Flavonoids	+	+	+
Tannins	+	+	+
Coumarins	+	+	+
Steroids	+	+	+

Keynote: (+) presence; (−) absence; *S. plumosum* (S), *S. pinnata* (SC), *T. cilliata* (T).
